# TOPICOP©: A New Scale Evaluating Topical Corticosteroid Phobia among Atopic Dermatitis Outpatients and Their Parents

**DOI:** 10.1371/journal.pone.0076493

**Published:** 2013-10-16

**Authors:** Leïla Moret, Emmanuelle Anthoine, Hélène Aubert-Wastiaux, Anne Le Rhun, Christophe Leux, Juliette Mazereeuw-Hautier, Jean-François Stalder, Sébastien Barbarot

**Affiliations:** 1 Public Health Department, University Hospital of Nantes, Nantes, France; 2 EA 4275, Biostatistics, Clinical Research and Subjective Measures in Health Sciences, Faculty of Pharmaceutical Sciences, University of Nantes, Nantes, France; 3 Dermatology Department, University Hospital of Nantes, Nantes, France; 4 Dermatology Department, University Larrey Hospital, Toulouse, France; Gentofte University Hospital, Denmark

## Abstract

**Background:**

The fear of using topical corticosteroids, usually called topical corticophobia, is a frequent concern for atopic dermatitis patients and/or their parents. Assessing patients’ atopic dermatitis and their parents’ topical corticosteroid phobia is an essential step to improving adherence to treatment. Because topical corticophobia appears to be a complex phenomenon, its evaluation by binary responses (yes/no) is too simplistic. Thus, a scale is needed, which is capable of identifying the subtleties of topical corticosteroid phobia.

**Objectives:**

To develop and validate a scale, TOPICOP©, measuring worries and beliefs about topical corticosteroids among atopic dermatitis outpatients and their parents.

**Methods:**

An initial statistical validation of TOPICOP was carried out, collecting qualitative data about patients’ topical corticophobia behaviors and beliefs using focus-group methodology. Then, 208 outpatients or their parents from five French centers completed a self-administered questionnaire built from focus-group results. The scale-development process comprised an explanatory principal component analysis, Cronbach’s α-coefficients and structural equation modeling.

**Results:**

The validated questionnaire comprised 12 items, covering two important dimensions relative to “worries” (6 items) and “beliefs” (6 items). Psychometric properties showed that items had very good communality (>0.60) within their own dimension. The final two-factor solution accounted for 47.3% of the variance. Cronbach’s α-coefficients were, respectively, 0.79 and 0.78. Structural equation modeling strongly supported the possibility of calculating a global score.

**Conclusions:**

TOPICOP© is the first scale aimed at assessing topical corticophobia in adult patients and parents of children with eczema. TOPICOP® has excellent psychometric properties and should be easy to use in everyday clinical practice for clinicians and researchers. Further studies are needed to confirm our results and validate TOPICOP© in other cultures.

## Introduction

Atopic dermatitis (AD) is a chronic inflammatory cutaneous disorder that often affects patients’ and parents’ quality of life [1]–[4]. AD is also a common public health problem, because of its increasing prevalence throughout the world, and its significant cost to society [5]–[8].

Topical corticosteroids (TCS) combined with emollients remain the mainstay of AD treatment. Their efficacy and safety, when appropriately used, has been clearly established [9], [10]. Paradoxically, the fear of using TCS (usually called TCS phobia, TCP) is a frequent concern for patients and their parents (between 40% and 73% depending on the authors) [4], [11]–[13]. This fear could be the main cause of poor therapeutic adherence and consequently poor treatment response for many patients (only 32% of AD patients seem to adhere to medical instructions) [14]. The evaluation of TCP is thus an essential step in the management of patients with poor adherence.

Few studies have addressed the assessment of TCP; most of them used only one question with a yes/no response [11], [12]. Because TCP appears to be a complex phenomenon, its assessment through binary responses is too simplistic, as it cannot detect different types of fear. Thus, a scale, which is capable of identifying the subtleties of this concept, is needed [15]. Although generic psychology scores exist to explore worries [16], [17], to the best of our knowledge, no such medical tool has been published to assess specific beliefs and worries related to TCS.

Herein, we describe the development and statistical validation of a scale named TOPICOP© (topical corticophobia) aimed at assessing AD outpatients’ and their parents’ worries and beliefs about TCS. This scale will help researchers and clinicians to better understand the factors that influence therapeutic adherence.

## Materials and Methods

### Study Design

This prospective multicenter study was conducted in five regions in France. A convenience sample was created with the help of 9 hospital dermatologists and 53 dermatologists in private practice. They gave a self-administered anonymous questionnaire to their consecutive AD patients consulting between February and May 2009. Patients were asked to complete the questionnaire before the consultation. The first page of the document was a written explanation of the study that had to be signed by the respondent for participation. In all cases, the patient’s dermatologist had confirmed the AD diagnosis. All patients with AD were included, and parents were asked to fill out the questionnaire for their affected children under 15 years old.

The local Ethics Committee of Nantes University Hospital approved this study.

### Questionnaire-construction Process

The first step of the questionnaire-construction process used focus-group methodology [18]. This qualitative phase, conducted between September and December 2008, involved 12 adult patients, nine parents of children with AD, and 15 health professionals (8 general practitioners and 7 pharmacists) in the collect of data on patients’ behaviors, beliefs, cognitions, sensations and perceptions of TCP. Five focus-group meetings were held and interviews were transcribed. In addition, four telephone interviews sufficed to reach the data saturation threshold. Analysis was completed by an examination of the literature. The qualitative phase was previously reported in details [15].

The second step enabled us to generate a 51-item questionnaire with an additional 18 items concerning sociodemographic characteristics and health status. To ensure content validity, a panel of seven experts (four dermatologists, one psychologist, and two public health doctors) discussed and retained the list of items. To ensure face validity, the questionnaire was given to a panel of 10 patients to explore the level of understanding, acceptability and time required to complete the questionnaire. Five items were modified slightly in response to patients’ comments.

Among the 51 items, 32 concerned the different types of worries and beliefs identified. The other items explored the origins of fears and beliefs (lack of oral or consistent information delivered by caregivers, discrepancies concerning treatment among dermatologists, dermatologists and general practitioners, and between practitioners and pharmacists, roles of family circle and the media…), behaviors and therapeutic adherence and, finally, the characteristics of the patients and their AD. Three items were eliminated from the questionnaire for scale construction because they were specific to children. For the 29 remaining items studied in this paper, response choices had a 4-point Likert-scale format (from totally disagree to do not really agree, almost agree or totally agree; or never to sometimes, often or always).

### Scale-construction Process (Construct, Concurrent and Convergent Validity, Reliability)

The first statistical analyses used the usual techniques of descriptive statistics (frequency, means ± SD) and Pearson’s correlation coefficients between items two-by-two.

The first step consisted of eliminating items with a rate of missing values >20%, or a floor or ceiling effect >50%. Then, when two of the remaining items had a Pearson correlation coefficient >0.60, one of them was selected in accordance with the consensus of the seven experts. The second step was an explanatory principal component analysis using a varimax rotation for the 19 remaining items. The number of dimensions was determined using a scree plot and clinical relevance. Two criteria were used to attribute each item to one of the dimensions: substantial communality (>0.60) with one principal component and, when an item exhibited communality across several dimensions, it was attributed to the one for which it maximized internal consistency assessed by Cronbach’s α-coefficient (>0.7). This strategy led to the removal of seven items, for which neither a sufficient communality with principal components nor an adequate Cronbach’s α-coefficient could be obtained, yielding a robust shorter two-dimension solution. Finally, the homogeneity of the dimensions was assessed using convergent validity (correlation of each dimension item with all the other items in the dimension >0.40), and divergent validity (correlation of each dimension item with all the other items in the other dimension <0.40) [19]. Overlap was corrected. To explore criterion validity, Pearson’s correlation coefficients were calculated between scores obtained and a visual analog scale evaluating the intensity of fears.

### Structural Equation Modeling Using the Linear Structure–relationship Approach

Structural equation modeling was performed to confirm factorial structure and unidimensionality of the dimensions. It is a comprehensive statistical approach to test hypotheses about relationships among observed and latent variables (dimensions) [20]. Several statistical indices were calculated in order to verify model fit and to select the best-suited model. Parameter estimation used the linear structural–relationships approach developed by Jöreskog [21]. To do so, the three main criteria used were Steiger’s root mean square error of approximation with the fit being considered good when <0.1 and very good when <0.05; Bentler and Bonnet’s normed fit index considered good when >0.9, and the goodness-of-fit index considered good when >0.9 [22].

### Score Calculation

Four response choices were offered, from totally disagree to totally agree, with points attributed to each one (0, 1, 2 or 3), with higher values corresponding to more severe TCP. Individual scores for all patients who responded to at least half the items plus one in a dimension were calculated by summing responses to items and then dividing that value by the number of items completed, yielding a maximal score of 36, expressed as a percentage. The mean score for a dimension was the sum of individual scores divided by the number of respondents. TOPICOP© scores ranged from 0 to 100.

All study analyses were computed using R 2.9 and SPAD 5.6. Structural equation modeling used SAS v9.1 (SAS Institute, Cary, NC) and its “PROC CALIS” procedure.

## Results

### Characteristics of the Sample

A total of 208 patients or parents were enrolled in the five French regions: hospital dermatologists enrolled 114 patients or parents and dermatologists in private practice enrolled 94. Among the 208 respondents, 144 were parents of children with AD. Mean ± standard deviation (SD) age of the respondents was 32.7±7.3 years. Concerning AD severity, 41.1% of patients or parents reported mild, 46.2% severe and 12.7% very severe disease. The majority of patients or parents (81.7%) were currently using or had recently used TCS. For a general item exploring fear about TCS, 80.7% admitted having fears about TCS.

### Scale-construction Process

**Figure 1:**
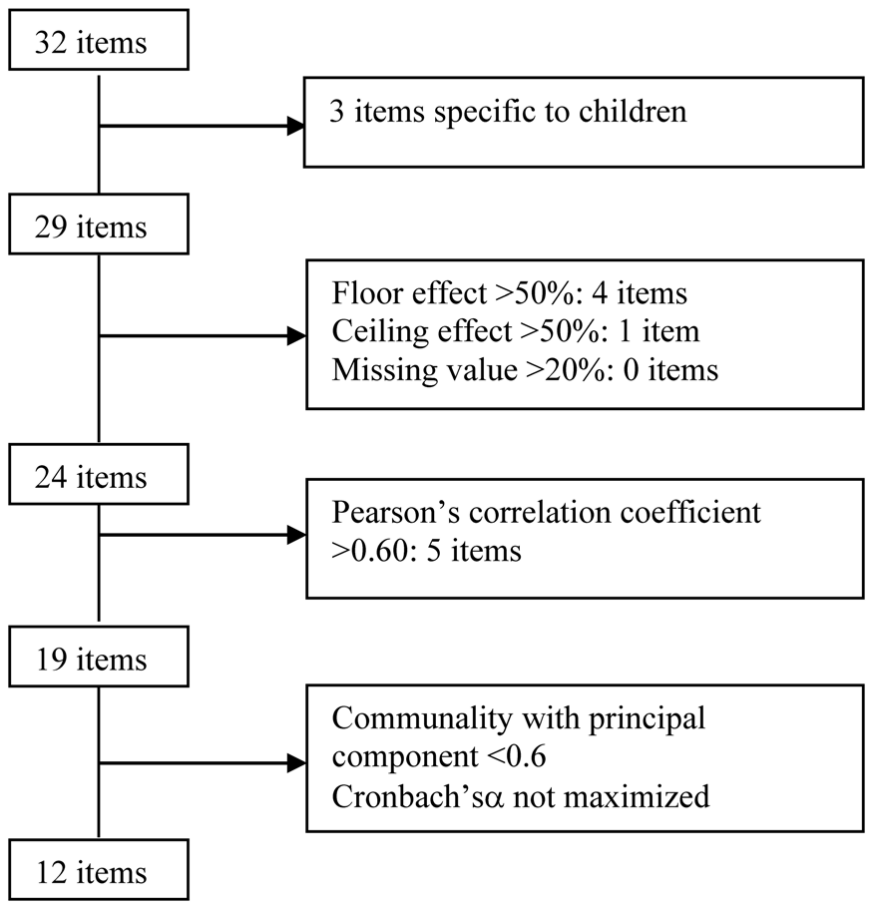
Selection process of items (flow chart).

Twenty-nine items exploring different types of worries and beliefs were studied (Fig. 1). No item had a missing-value frequency >20%. A floor effect (percent of ‘totally disagree’ responses exceeding 50%) was observed for four items and one had a totally agree percentage >50% (ceiling effect): they were excluded. Among the 24 remaining items, five were removed whose Pearson’s correlation coefficients with another item exceeded 0.6. For example, the correlation coefficients of the two following items “I’m afraid of applying too much cream” and “I’m afraid of using the cream for too long” were 0.75. The expert panel decided to retain the former item. Successive explanatory principal component analyses were then performed on the 19 remaining items and led to the identification of two dimensions. Seven items were removed step-by-step because of their low communality with one of the two factors or because they exhibited communality with both. No item maximized Cronbach’s α-coefficient and all the 12 remaining items had communality >0.60 within their own dimension.

The final questionnaire TOPICOP©, a 12 item scale (Table 1), accounted for 47.3% of the variance, with a first dimension of six items exploring worries (WOR) accounting for 24.4% of the variance, and a second one, also of six items exploring beliefs (BEL) accounting for 22.9% of the variance; their respective Cronbach’s α-coefficients were 0.79 and 0.78. Correlations between items within a given dimension all exceeded 0.40 and correlations between one item and those of the others in the dimension were <0.40. Inter-dimension correlation was 0.41. Pearson’s correlation coefficient between the WOR dimension and visual analogue scale score exploring fears was 0.60. The statistical parameters are reported in Table 2. The final questionnaire is presented in Table 3.

**Table 1:** TOPICOP© scale: results of principal component analysis using Varimax rotation according to dimension.

		Dimensions	
Dimension	Item^1^	WOR	BEL
BEL1	TCS pass into the bloodstream	0.14	**0.61**
BEL 2	TCS can lead to infections	0.15	**0.68**
BEL 3	TCS make you fat	–0.04	**0.62**
BEL 4	TCS damage your skin	0.18	**0.70**
BEL 5	TCS will affect my future health	0.29	**0.72**
BEL 6	TCS can lead to asthma	0.12	**0.61**
WOR1	I don’t know of any side effects but I’m still afraid of TCS	**0.66**	0.17
WOR 2	I’m afraid of applying too much cream	**0.72**	0.21
WOR 3	I’m afraid of putting cream on certain zones like the eyelids, where the skin is thinner	**0.67**	0.17
WOR 4	I wait as long as I can before treating myself	**0.62**	0.03
WOR 5	I stop the treatment as soon as I can	**0.61**	0.19
WOR 6	I need reassurance about TCS	**0.76**	0.06

1 The 12 items are those of the TOPICOP© scale. Response choices were accorded 0–3 points (Likert scale) corresponding to totally disagree to totally agree (BEL1 through WOR 1) and never to always (WOR 2 through WOR 6).

Abbreviations: WOR, worries; BEL, wrong beliefs.

**Table 2:** Psychometric properties of the TOPICOP©, according to the worries (WOR) and beliefs (BEL) dimensions.

	Dimensions		
Item property	WOR	BEL	TOPICOP©
*Descriptive*			
Items in the scale, n	6	6	12
Questionnaires with at least 50% +1 items completed, n	205	205	206
Questionnaires with 50% +1 items completed, n	99.0%	99.0%	99.5%
Items with “missing data” >20%, n	0	0	0
Items with “does not apply” response >20%, n	0	0	0
Items with ceiling effect >50%, n	0	0	0
Items with floor effect >50%, n	0	0	0
Mean score (standard deviation), n	46.4 (24.7)	41.0 (22.1)	43.9 (19.6)
Skewness value/standard error	0.06/1.54	0.24/1.54	0.18/1.36
Median	44.17	44	44
*Statistical*			
Ceiling effect	0.49%	0.49%	0%
Floor effect	2.44%	2.44%	0%
Items whose correlation within own dimension >0.40, n	6	6	–
Items whose correlation within own dimension greater than with other scale, n	6	6	–
Inter-scale correlation	–	–	0.41
Cronbach’s α-coefficient	0.79	0.78	0.81
Dillon–Goldstein ρ-coefficient	0.85	0.84	0.85
Sum of squares of the dimension before rotation	33.2%	14.1%	47.3%
Variance explained by the dimension	24.4%	22.9%	47.3%
First eigenvalue	2.88	2.75	3.99
Second eigenvalue	1.04	0.82	1.69

Abbreviations: WOR, worries; BEL, wrong beliefs.

**Table 3:** Items of the TOPICOP©.

**TCs pass into the bloodstream**				
	Totally disagree	Not really agree	Almost agree	Totally agree
**TCs can lead to infections**				
	Totally disagree	Not really agree	Almost agree	Totally agree
**TCs make you fat**				
	Totally disagree	Not really agree	Almost agree	Totally agree
**TCs damage your skin**				
	Totally disagree	Not really agree	Almost agree	Totally agree
**TCs will affect my future health**				
	Totally disagree	Not really agree	Almost agree	Totally agree
**TCs can lead to asthma**				
	Totally disagree	Not really agree	Almost agree	Totally agree
**I don’t know of any side effects but I’m still afraid of TCs**				
	Totally disagree	Not really agree	Almost agree	Totally agree
**I’m afraid of applying too much cream**				
	Never	Sometimes	Often	Always
**I’m afraid of putting cream on certain zones like eyelids, where the skin is thinner**				
	Never	Sometimes	Often	Always
**I wait as long as I can before treating myself**				
	Never	Sometimes	Often	Always
**I stop the treatment as soon as I can**				
	Never	Sometimes	Often	Always
**I need reassurance about TCS**				
	Never	Sometimes	Often	Always

### Structural Equation Modeling with the Linear Structure–relationship Approach

**Figure 2:**
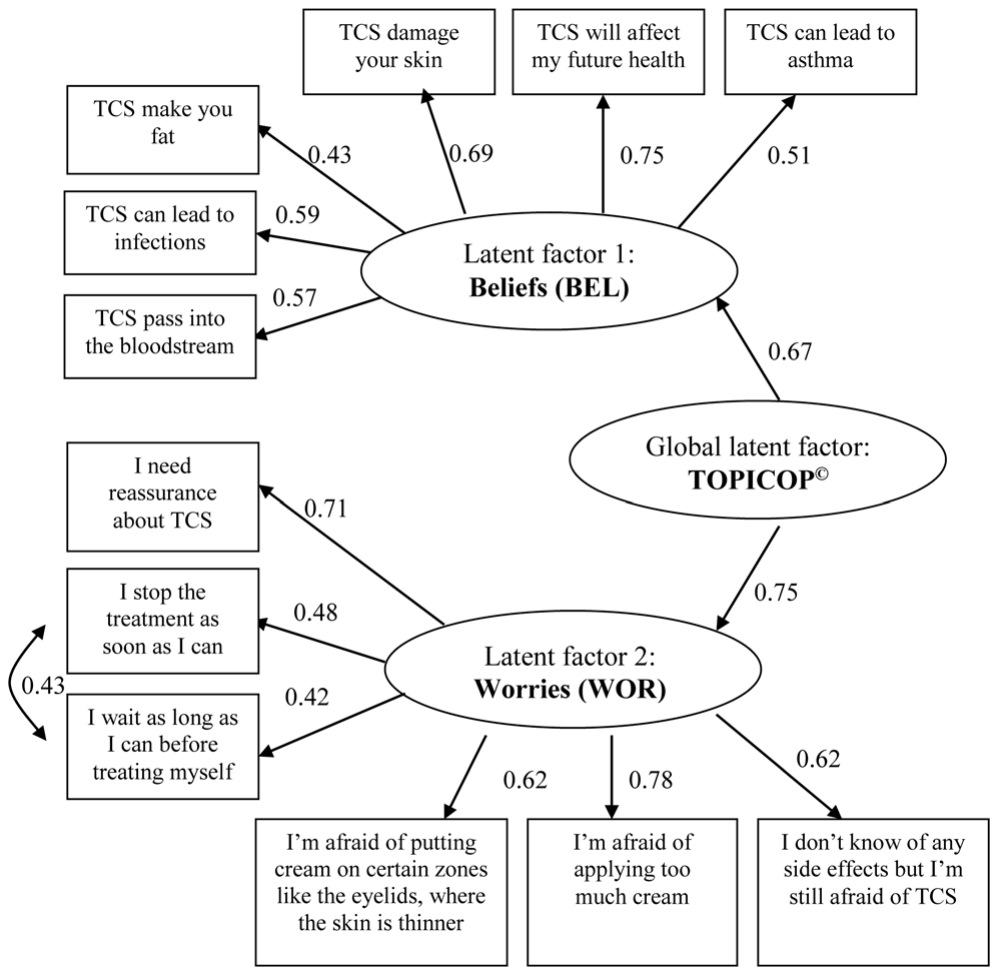
Structural equation model of the TOPICOP© scale. Abbreviation: TCS, topical corticosteroids. Goodness-of-fit criteria: Steiger’s root mean square error of approximation = 0.005; Bentler and Bonnet’s normed fit index = 0.89; Goodness-of-fit index = 0.94.

Modeling confirmed the existence of two latent dimensions WOR and BEL but the best characteristics were obtained with a hierarchical model including the two latent factors and a global latent factor, bringing the 12 items together (Fig. 2). Goodness of fit of the data were very good and all the structural coefficients were highly significant (*P*<0.001).

### TOPICOP© Mean Scores

Based on scores ranging from 0 to 100, mean (±SD) scores were 46.4±24.7 for WOR dimension, 41.1±22.1 for BEL dimension and 43.9±19.6 for the TOPICOP© scale. Results are reported in Table 2.

## Discussion

TCP is a common, worldwide phenomenon in patients with atopic dermatitis, leading to poor local treatment adherence and frequent therapeutic failure. Paradoxically, there is a lack of TCP assessment tools available. We previously showed that TCP is a complex phenomenon [15]: some patients who did not admit to being worried about TCS expressed TCS phobia through their behaviour (need for reassurance or reducing doses). Assessing TCP with binary (yes-or-no) responses is, thus, too simplistic and cannot detect different types of fears and their intensity. The need to develop a scale aimed at assessing the multidimensional aspects of TCP (worries, beliefs and behaviours) is proven.

TOPICOP© is the first self-administered scale, developed to evaluate AD patients’ or their parents’ TCP, to have undergone initial statistical validation. It contains 12 items; six of them in each of the two dimensions related to worries and beliefs (WOR and BEL), and exhibits excellent psychometric properties. The statistical validation strategy presented herein follows most of the recommendations of ‘good practice’ for score validation [23].

The first original feature of our study was the methodology used to develop the questionnaire. Item generation, based on a qualitative analysis using focus groups, enabled us to design a questionnaire that explored the AD patients’ or parents’ real-life attitudes, worries, beliefs and behaviors [15]. This first step enabled us to ensure good content and face validity. The second step consisted of a quantitative study derived from the conclusions of the former and enabled us to statistically validate the TOPICOP© scale. This step supported high construct, divergent and discriminant validity and reliability. The small numbers of missing values indicate a very good understanding of the questionnaire. Internal consistency was close to 0.80 as recommended [24], [25]. Items had strong communality with the two principal component analysis-identified factors and accounted for >47% of the variance. To confirm our results, structural equation modeling was performed and strongly upheld the possibility of calculating a global score. As TCP is mainly a parents’ problem in daily practice, our population was made up of two thirds parents and one third adult patients. We cannot exclude a recruitment bias. As we recommended to give the questionnaire to consecutive patients with AD, it might have been distributed to those with more severe AD or with TCS phobia. Finally, to reduce the recruitment bias, parents and adult patients were enlisted from both hospital outpatient-dermatology departments and private dermatology practice. In France, patients consulting at hospital departments might have more severe disease or more TCS worries leading to therapeutic failure.

Nevertheless, despite TOPICOP©’s excellent psychometrics properties, a question remains: is it able to represent the reality of the TCP concept? Corticophobia first appeared 25 years ago in the context of asthma [26], [27] and is now recognized as a very common but poorly understood phenomenon [28]. In dermatology, TCP is characterized by patients’ fear and excessive anxiety about using TCS preparations. Use of the term “phobia” seems to be a little excessive with regard to its psychiatric definition as, according to psychiatrists, a phobia is an intense but unrealistic fear that can interfere with the ability to socialize, work or go about everyday life, brought on by an object, event or situation. A specific phobia is the fear of a particular situation or object [29], [30] but worries about TCS are not always unfounded. For example, it is true that TCS can pass into the blood stream or can damage the skin by causing permanent thinning and vasoplegia. The question, then, is not to qualify a patient’s worries and beliefs about TCS as being true or false, but rather to understand to what extent those worries and beliefs have an impact on adherence to treatment. Because worries and beliefs are linked, the TOPICOP© scale comprises items from these two facets of TCP in AD.

Items selected concerning beliefs (cutaneous side effects; infections; systemic side effects, principally growth retardation, weight gain or inducing asthma) are consistent with those mentioned earlier by numerous authors [11], [12], [31]. Items related to worries (TCS dependency or addiction, loss of efficacy, need for reassurance) have also been reported [11], [12], [32]. Furthermore, many patients in our study said they did not know the side effects of TCS but were still afraid of using them, highlighting that negative beliefs and attitudes concerning TCS were not always based on scientific findings [11], [15]. Their mean TOPICOP© scale scores demonstrated that TCP is an important and frequent problem in AD treatment, with behavioural consequences that lead to poor local treatment adherence in AD patients or parents and therapeutic failures.

Even though the question of patient attitudes to corticosteroids is frequently asked in consultation, the origins of these fears are rarely explored. In fact, there can be a variety of reasons for this reticence: personal experience, divergent advise from pharmacists and doctors, divergent advise from friends and family, information found on the internet etc. The TOPICOP scale should thus help clinicians to better identify their patients’ fears and worries relating to TCS in order to personalize their discourse, target specific blockages and develop pertinent arguments to help patients/parents to adhere to prescribed treatment. Nevertheless, our study was not designed to assess the potential impact of using TOPICOP© scale on patients’ adherence.

Furthermore, The TOPICOP© scale should help researchers assess and explore TCP in clinical studies. Indeed, as TCP is closely linked to therapeutic adherence in AD, we suggest that it should be systematically assessed in clinical trials as a potential factor influencing outcomes. Moreover, TOPICOP© scale should be useful in future studies to evaluate to what extent patient education could modify patients’ TCP thresholds. TOPICOP© also covers one of the main research topics proposed by the HOME study group [33]. Finally, the items included in TOPICOP© were chosen according to the oral depositions of patients during the focus group meetings. The items are thus simple and easily understood.

TOPICOP© was created and tested in France but the dimensions explored by the scale are not limited to French AD patients and further scale validation in other countries and cultures is required in order to facilitate international comparative studies. These international TOPICOP© versions need to be explored in terms of cross-cultural adaptation and necessitate an accurate translation combined with posteriori verification by a medical expert. Nevertheless, an English version of the TOPICOP© scale is already available, translated by a native English-speaking American scientist and reviewed by our panel of experts.
